# Protocol for a multi-centre randomised controlled stand-alone feasibility trial to assess potential effectiveness and cost-effectiveness of digital hearing aids in patients with tinnitus and hearing loss (the HUSH trial)

**DOI:** 10.1186/s40814-020-00582-5

**Published:** 2020-03-27

**Authors:** Rachel H. Haines, Jennifer White, Garry Meakin, Wei Tan, Trish Hepburn, Paul Leighton, Chloi Theriou, David Stockdale, Christine Almey, Richard Nicholson, Deborah A. Hall, Magdalena Sereda

**Affiliations:** 1 Nottingham Clinical Trials Unit, University of Nottingham, Nottingham, UK; 2 Centre of Evidence Based Dermatology, University of Nottingham, Nottingham, UK; 3 National Institute for Health Research Nottingham Biomedical Research Centre, Nottingham, UK; 4 British Tinnitus Association, Sheffield, UK; 5 PPI Representative, Leicester, UK; 6 Nottingham University Hospitals NHS Trust, Nottingham, UK; 7 Hearing Sciences, Division of Clinical Neuroscience, School of Medicine, University of Nottingham, Nottingham, UK

**Keywords:** Protocol, Randomised controlled trial, Feasibility, Tinnitus, Hearing loss, Hearing aids, Outcomes

## Abstract

**Background:**

The most common management strategy for tinnitus provided in the UK audiology clinics is education and advice. This may also be combined with some form of sound therapy (e.g. digital hearing aids). While education and advice is generally provided by all clinics, there is a marked variability in provision of hearing aids that depends very much on clinical decisions. A recent Cochrane review concluded a lack of evidence to support or refute hearing aid use as a routine intervention for people with tinnitus and hearing loss. This lack of evidence is reflected in the inconsistency of tinnitus management in the UK. The aim of the HUSH trial is to determine the feasibility of conducting a definitive randomised controlled trial (RCT) of the effectiveness and cost-effectiveness of hearing aids for adults with tinnitus and hearing loss.

**Methods:**

This is a multicentre randomised controlled feasibility trial. Up to 100 adults, aged 18 and over, presenting to 5 UK audiology clinics with a complaint of tinnitus and measurable hearing loss are being randomised to receive either (i) education and advice (treatment as usual) or (ii) education and advice with digital hearing aids. Feasibility outcomes are being collected around recruitment, retention, patient and healthcare professional acceptability and clinical outcome assessment. Outcomes are being collected via postal questionnaire at 12 weeks post baseline. A nested interview study will supplement clinical and other outcome data, providing a detailed understanding of participants’ and audiologists’ experience of both tinnitus management and the research processes.

**Discussion:**

This feasibility trial will help us to (i) determine if it is feasible to conduct a multicentre RCT comparing treatment as usual and treatment as usual plus digital hearing aids; (ii) optimise the design of a future definitive, multicentre RCT; and (iii) inform which outcome(s) is/are relevant for patients. This work presents an important first step in determining the effectiveness of hearing aids as a tinnitus management strategy.

**Trial registration:**

ISRCTN, ISRCTN14218416. Registered on 30 July 2018.

## Background

Tinnitus is defined as the perception of sound in the absence of an external source [1]. It is typically described by those who experience it as a ringing, hissing, buzzing or whooshing sound and is thought to result from abnormal neural activity at some point or points in the auditory pathway, which is erroneously interpreted by the brain as sound.

Tinnitus is a major problem. When defined as lasting for more than 5 min at a time, it has been shown to affect 12–30% of the adult population, with 3 to 31% reporting bothersome tinnitus [2]. Age and hearing loss are both known pre-disposing factors. For example, the incidence of clinically bothersome tinnitus rises with increasing age [3], and it is estimated that tinnitus prevalence in people with hearing loss may reach 70–85% [4–6]. Many people with tinnitus experience symptoms that negatively affect the quality of life (including sleep disturbances, hearing difficulties, difficulties with concentration, social isolation and emotional difficulties including anxiety, depression, irritation or stress) and require clinical intervention [5].

Tinnitus represents a significant burden on healthcare services. In England, it is estimated that there are ¾ million primary care consultations per year with a primary complaint of tinnitus [7]. In the UK, care pathways include appointments with Ear Nose and Throat (ENT) and audiology specialists (in all combinations) [8]. Yet, currently, there are no National Institute for Health and Care Excellence (NICE) clinical guidelines for the management of tinnitus. The Department of Health Good Practice Guide [9] does provide some guidance on the commissioning of tinnitus services in the National Health Service (NHS). However, this is not evidence-based and it lacks specific recommendations on the provision of hearing aids for patients with tinnitus and hearing loss.

In the UK, the most common management strategy is education and advice. This typically includes, but is not limited to, explanations of tinnitus and its association with hearing loss, education about lifestyle factors that may have positive or negative effects on tinnitus, and a description of available management strategies [10]. Some audiologists may also combine education and advice with some form of sound therapy, which is the provision of electronic devices to amplify external sounds (e.g. hearing aids) or to produce sounds for therapeutic use (e.g. sound generators) [9]. While education and advice is generally provided by all clinics, there is a marked variability in the provision of devices (i.e. hearing aids) that depends very much on local or individual clinical decisions [11].

The primary function of a digital hearing aid is to amplify and modulate sound to make the sound more accessible and to aid communication. Using hearing aids in tinnitus management has been proposed as a useful strategy since the 1940s [12], although the benefit reportedly varies and there is no clear consensus on when a person would or would not benefit from amplification [13, 14]. A number of ways in which hearing aids may be beneficial for tinnitus have been suggested [15]. Hearing aids can amplify environmental sounds and mask or provide a distraction from tinnitus. They can reduce listening effort and improve communication, which in turn can reduce the stress and anxiety commonly associated with tinnitus. Other possible mechanisms include physiological effects on tinnitus-related brain activity by ‘recalibrating central gain’ or preventing maladaptive plastic changes in the brain related to hearing loss. Alternatively, amplification may simply refocus attention on alternative auditory stimuli that are incompatible and unrelated to the tinnitus sound.

Despite these findings, there is no robust evidence that shows that hearing aids are more effective in alleviating tinnitus than education and advice alone. The Cochrane review of sound therapy for the management of tinnitus looked at the quality of current evidence of effectiveness of different forms of sound therapy for tinnitus [15]. There was no evidence to support the superiority of any form of sound therapy (including hearing aids) for tinnitus over waiting list control, placebo or education/information with no device. The review concluded that there is a lack of evidence to support or refute hearing aid use as a routine intervention for people with tinnitus and hearing loss. It called for further research to generate high-quality evidence for the effectiveness of different sound therapy options (including hearing aids) using rigorous methodology.

In line with the above, there is no standard care pathway for patients presenting with a complaint of tinnitus. Decisions are influenced by the experiences or personal opinions of individual audiologists, such that patients currently get different care depending on which audiologist they see. A European review of clinical guidelines observed that differences include recommendations for sound therapy as a form of treatment for tinnitus distress [16]. A survey of UK audiology departments has shown that half of the clinicians have different candidacy criteria for fitting hearing aids for patients with and without tinnitus [11, 13, 17]. Patients with mild hearing loss and tinnitus are less likely to receive hearing aids as are those who do not report hearing difficulties, despite the fact that tinnitus annoyance can be as debilitating in people who have mild or more severe hearing loss as in those who do not report hearing difficulties [18–20]. Evaluating the effectiveness of digital hearing aids for tinnitus relief is known to be a priority research question for clinicians and patients as this was identified in the top ten outcomes from a James Lind Alliance (JLA) Tinnitus Priority Setting Partnership [21].

The overarching objective of the present trial is to investigate the feasibility and acceptability of conducting a randomised controlled trial (RCT) comparing (i) education and advice (Treatment as Usual (TAU)) and (ii) TAU plus digital hearing aids, for the management of people with tinnitus and hearing loss.

To achieve this, objectives around recruitment, outcome assessment and acceptability have been defined. Addressing these objectives will (i) determine if it is feasible to conduct a multicentre RCT comparing TAU and TAU plus digital hearing aids; (ii) help to optimise the design of a future definitive, multicentre RCT; and (iii) inform which outcome(s) is/are most relevant for patients to guide the selection of a primary outcome for the definitive RCT.

## Methods

**Fig. 1:**
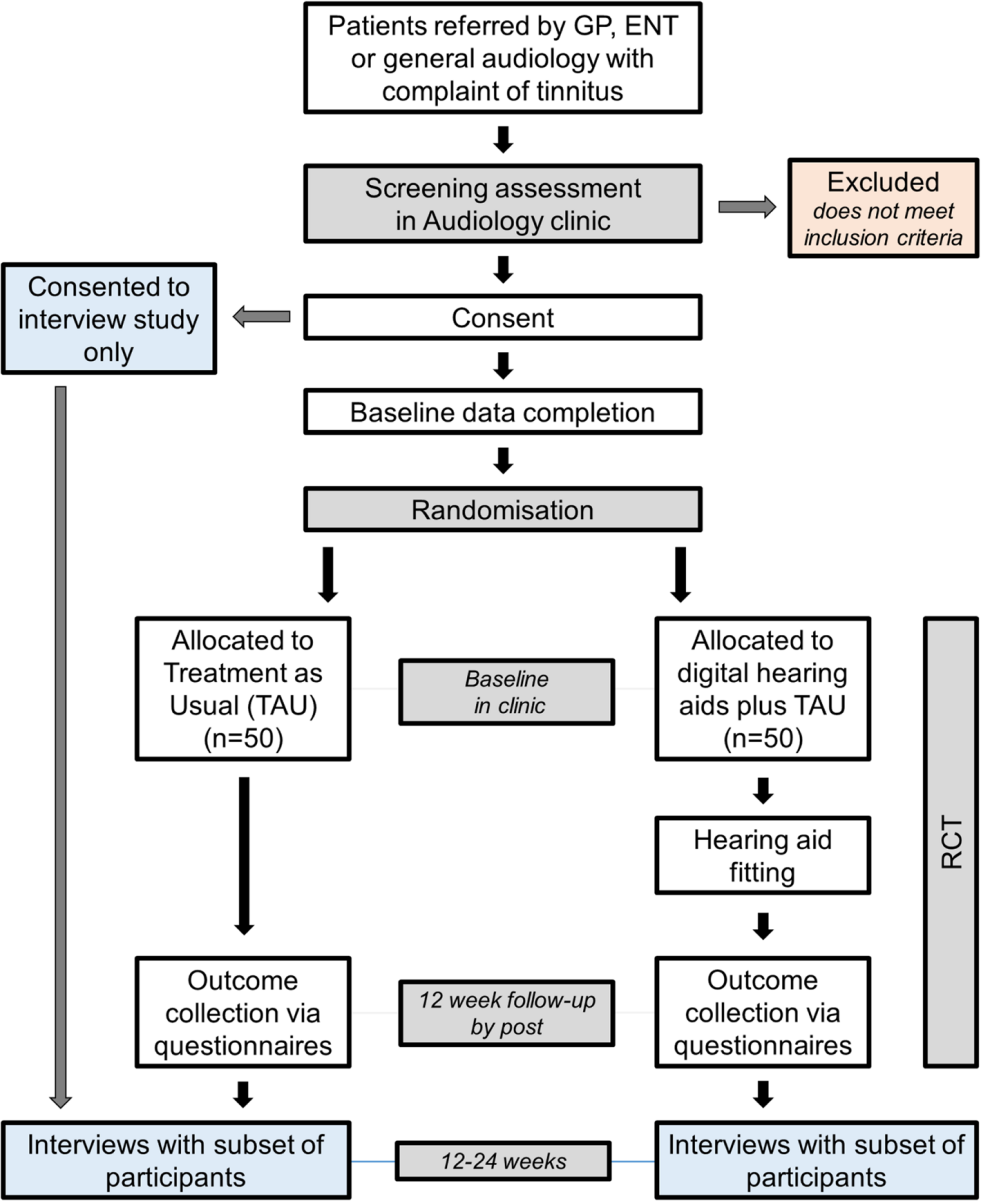
Flowchart indicating participant flow through the trial

This trial is an open-label, two-arm, multicentre, randomised controlled feasibility trial with an economic evaluation and a nested interview study (Fig. 1).

### Setting and participants

Five NHS audiology departments representing a range of clinical settings and service sizes are recruiting to the trial. The participants are adults, aged 18 and over, presenting to the audiology clinic with a primary complaint of tinnitus. Depending on local care pathways, participants may have been referred by their GP or an ENT department. All participants must be willing to provide written informed consent and have a clinical diagnosis of tinnitus, with hearing loss, as defined by the assessing audiologist. Potential participants will be excluded on the basis of the following: tinnitus of a medically treatable origin, inability to communicate in written English, having started or stopped medication for anxiety/depression within the last 3 months, previous use of a hearing aid in the last 12 months or current use of any combination device or behind the ear sound generator. A combination device is a digital hearing aid that also functions as a sound generator.

### Intervention

Both groups receive TAU, which, depending on local practice, may consist of any combination of the following: explanations of tinnitus and its association with hearing loss, education about lifestyle factors that may have positive and negative effects on tinnitus and explanation of available management strategies and different levels of counselling depending on patient need. For the purposes of the trial, TAU must not include the prescription of any combination device or at-ear sound generator and, for the control arm, must not include the provision of hearing aids.

In addition to TAU, the intervention group is also fitted with a digital hearing aid according to standard procedure. The hearing aid used must be CE marked and approved for use in the NHS and by local site for its intended purpose. The fitting audiologist should make a clinically appropriate daily usage recommendation, which is recorded by the audiologist using pre-defined categories (< 1 h per day, ≥ 1 to < 4 h per day, ≥ 4 to < 8 h per day, ≥ 8 h per day). Adherence is checked by comparing participant reported usage at 12 weeks to the audiologist recommended usage.

### Outcomes

Outcomes for the feasibility trial have been separated into those that determine the feasibility and acceptability of a large definitive trial and patient-reported outcome measures (PROMs). As the core outcome set for tinnitus is still under development [22, 23], there is no consensus on which PROM should be used as the primary outcome in a definitive trial. Therefore, in this feasibility trial, a range of PROMs have been used to inform the choice of the primary outcome for the definitive trial.

#### Feasibility outcomes

• Recruitment: Outcomes to assess whether it would be possible to recruit to a definitive trial.

- ▪ Proportion of patients eligible for the trial, patient and clinician views of the treatment options and barriers to screening and recruitment, number and proportion of patients recruited and randomised, characteristics of recruited patients, hearing aid adherence, trial retention, completeness of the collected data, components of TAU used across sites, hearing aid fitting parameters, mechanisms to capture re-referral rates.

• Acceptability: Outcomes to assess whether participants and clinicians would find the interventions and trial design acceptable.

- ▪ Feedback from participants and clinicians regarding experience with the trial (e.g. conduct, design, treatments) and opinions of the intervention.
- ▪ Hearing aid adherence

• Outcome assessment: Outcomes to help identify the most appropriate primary outcome for a definitive trial.

• Participant opinion of relevance of self-reported outcome measures; Distribution of patient-reported outcome measures; estimation of clinically important differences

• Safety: Capturing exacerbation of symptoms and adverse event monitoring to assess the feasibility of safety data capture for a definitive trial.

• Healthcare resource use questionnaire to assess the feasibility of capturing health economic data

#### Patient-reported outcome measures

All PROMS are collected at baseline and 12 weeks to measure changes between the two time points in the following:

• Tinnitus symptom severity, measured with the Tinnitus Functional Index (TFI) [24]

• Effects of hearing impairment on emotional and social adjustment, measured with the Hearing Handicap Inventory for the Elderly (HHIE) [25]

• General health status and health-related quality of life, measured with the Health Utilities Index Mark 3 (HUI3) [26]

• Depression and anxiety, measured with the Hospital Anxiety and Depression Scale (HADS) [27]

• The outcomes that the patient considers the most important on one or two symptoms related to tinnitus that they are seeking help with, measured with the Measure Yourself Medical Outcome Profile (MYMOP2) [28]

• Health-related quality of life, measured with the EQ-5D-5L [29]

• Participant-reported improvement in tinnitus and hearing (Global Rating of Change Score): measuring participant reported change in tinnitus using a 7-point Likert scale to the questions: (1) All things considered, how is your overall tinnitus condition now, compared to 3 months ago? (2) All things considered, how is your overall hearing now, compared to 3 months ago? For both questions, response options are ‘much improved’, ‘moderately improved’, ‘slightly improved’, ‘no change’, ‘slightly worse’, ‘moderately worse’ or ‘much worse’.

### Randomisation allocation, concealment and blinding

Randomisation (1:1 ratio) to the two trial arms uses a minimisation algorithm, minimising by trial site, tinnitus symptom severity and degree of hearing loss. Tinnitus symptom severity is categorised on three levels informed by TFI data acquired in the baseline clinic appointment (mild 0–28, moderate 29–65, severe ≥ 66 [30]), and the degree of hearing loss is categorised on two levels based on pure tone average of air-conduction thresholds at 0.5, 1, 2, 4, 6 and 8 kHz (≤ 40 dB, > 40 dB), measured no more than 6 months prior to the date of randomisation.

### Study procedures

Potential participants are screened from upcoming tinnitus clinic lists and the participant information sheet is sent in the post. Further eligibility screening takes place at the beginning of the routine care appointment, and the audiologist approaches the patient during their clinic visit if they are eligible for the trial. If written informed consent is provided after speaking further with the audiologist, site staff randomise the individual participant via a secure Web-based electronic randomisation system developed and maintained by the Nottingham Clinical Trials Unit (NCTU). Allocation determines which treatment(s) the clinician proceeds to offer the participant during the routine care appointment. If the participant is randomised to the intervention arm, the hearing aid may be fitted at a separate appointment (up to 4 weeks post randomisation).

During the 12-week treatment phase, participants may withdraw from the trial intervention but remain in the trial for the purpose of providing follow-up data only. All participants receive a questionnaire pack in the mail at 12 weeks post randomisation for the collection of trial outcomes. Participants have a 1-month window to return the completed questionnaire. To maximise retention, trial management staff start to chase non-completed questionnaires around 2 weeks after first sending. Questionnaire reminders are communicated to participants via post, email, text and telephone. Trial participants re-integrate the local standard care pathway upon conclusion of their involvement in the trial and may request to be reassessed at that time by their local clinic.

The structure of the trial and the timing of enrolment and assessments are detailed in Table 1. Audiometry and TFI must be collected prior to randomisation to enable stratification.

**Table 1:** Summary of assessments

	Trial period		
	Baseline	12-week follow-up	Participant interviews (within 1 month of follow-up completion)
**Enrolment**			
**Eligibility screen**	X		
**Informed consent**	X		
**Randomisation**	X		
**Assessments**			
**Baseline characteristics**	X		
**Audiometry**	^a^X		
**Hearing aid specification**	^b^X		
**Hearing aid fitting**	^c^X		
**TFI**	X	X	
**HHIE**	X	X	
**HUI3**	X	X	
**HADS**	X	X	
**MYMOP2**	X	X	
**EQ-5D-5L**	X	X	
**Healthcare resource use**	X	X	
**Hearing aid adherence**	X	X	
**Safety reporting**		X	
**Global rating of change score**		X	
**Experience of tinnitus management**			X^de^
**Experience of research process**			X^de^

^a^Hearing thresholds measured no more than 6 months prior to baseline may be reused

^b^For those randomised to receive a hearing aid

^c^May take place at baseline or at a second fitting appointment. Fitting should take place ideally within 2 weeks up to a maximum of four from the time of the first visit

^d^A subset of participants and audiologists only

^e^For patients who consent to taking part in the interviews only, interviews for those selected will take place within 1 month of consent. Audiologist interviews will take place towards the end of the recruitment period of the trial

### Blinding

Blinding of treatment allocation is not possible for the clinician or the participant, as hearing aids are offered only to the intervention group. The trial statistician, health economist and chief investigator are blinded to all treatment allocations. The trial management staff are also unblinded to treatment allocation in order to send out the appropriate version of the 12-week follow-up questionnaire.

### Data collection and management

All data and participant contact details are treated confidentially and held on two separate secure University of Nottingham servers with restricted and password-protected access. Data checks are undertaken in line with the data management plan, and the manual entry of all audiogram data is centrally crosschecked with a screenshot of the audiograms to ensure accurate entry.

Regular central monitoring activities are undertaken by the NCTU, and sites are monitored in accordance with the data monitoring plan. Audits will take place at the request of the sponsor or regulatory authorities.

### Sample size

Since this is a feasibility trial, a formal calculation was not appropriate. The trial aims to randomise 100 participants over 12 months from five recruiting centres to explore feasibility parameters such as methods of recruitment and recruitment and retention rates in different types of centres. This sample size will enable estimation of a retention rate of greater than 80% to within 8% points of the true value with 95% confidence. Together with information from qualitative interviews and the information collected about PROMs which will inform the primary outcome and sample size for a future trial, this should provide sufficient to determine feasibility for a future definitive trial.

### Planned analyses

For this feasibility trial, there are no stopping rules defined or formal interim analysis planned. The planned final analysis will take place when all data relating to all outcomes have been collected, the database has been locked and the treatment codes revealed.

The analysis and reporting of the trial will be in accordance with the Consolidated Standards of Reporting Trials (CONSORT) [31] and the CONSORT extension for randomised pilot and feasibility trials [32]. A full statistical analysis plan (SAP) will be developed and finalised prior to database lock. Following the CONSORT guidelines, a flow diagram showing the numbers of people referred, approached and screened, eligible, consented and randomised (with reasons for exclusions) will be produced.

Recruitment rates at sites will be summarised using appropriate statistics. Completeness of data collection at baseline and 12 weeks will be summarised by trial arms. Descriptive summaries of outcome data at baseline and 12 weeks will be presented. Formal statistical testing for between-arm comparisons will not be performed; however, differences between arms may be presented with 95% confidence intervals where appropriate. The number and proportion of participants who underwent each allocated procedure will also be summarised by trial arms.

### Analyses for selection of potential primary outcome

Investigation of PROMs will be performed independently of the treatment group. The total scores at baseline and 12 weeks will be summarised for the five PROMs (TFI, HHIE, HUI3, HADS and MYMOP2) listed in the ‘Patient-reported outcome measures’ section. The change from baseline for PROM scores will be calculated and summarised at 12 weeks.

A global improvement question will be answered at 12 weeks. PROM scores for participants who answered “slightly improved” or “moderately improved” will be used as an anchor to investigate the smallest difference that the participants perceive to be beneficial.

Minimum clinically important effects for each PROM will be estimated using three anchor-based responsiveness statistics: (i) standardised response mean (SRM), (ii) effect size (ES) and (iii) Guyatt’s Responsiveness Index (GRI). This analysis, along with patients’ views on the relevance of PROMs to their tinnitus (captured as a part of the questionnaires and nested interviews), will guide the choice of relevant PROMs for use in a definitive trial of treatment of tinnitus.

### Economic evaluation

An exercise in health economic evaluation is being undertaken in the trial. The objectives of this work are to (i) determine whether key resource use and health-related quality of life (HRQoL) can be obtained from data collected remotely from participants, (ii) identify unit costs from secondary sources to attach to the resource use, (iii) design and test a cost pro forma for participants to complete, (iv) execute the steps necessary for a cost-utility analysis to determine whether TAU combined with digital hearing aids is likely to produce a feasible cost-effectiveness analysis and (v) explore the variables that indicate uncertainty.

The economic evaluation will explore the feasibility of collecting HRQoL and resource use data. The HUI3 was administered before and after the intervention for the calculation of HRQoL.

Data analysis for a preliminary cost analysis will be performed to explore incremental cost differences between the two trial arms. Quality-adjusted life-years (QALYs) with available data will be determined using the ‘area under the curve’ approach. This data will be explored in relation to costs.

### Stakeholder interview study

A nested interview study is being conducted alongside the feasibility trial. The purpose of this study is to (i) assess feasibility and acceptability of trial processes, (ii) assess feasibility and acceptability of the intervention (for both participants and clinicians) and (iii) to consider patient-reported outcome from the perspective of trial participants. Information will be gathered in a series of semi-structured interviews, which will be undertaken face-to-face or via telephone depending on the participants’ preference. Trial participants will consent separately for the interview study.

Approximately 30 participants will be interviewed after their 12-week treatment period has passed. Participants will be selected purposively to capture a range of different experiences and treatment outcomes. A small number of interviews (*n* < 10) will also be carried out with those who declined to take part in the trial to explore barriers to recruitment. These interviews will take place within 1 month of being approached about trial participation. After recruitment activities have ceased, approximately 10 clinical staff will be interviewed to review their experience of the trial in terms of participant recruitment and integrating the research within clinical care.

With the participants’ consent, data will be recorded and transcribed in full. Otherwise, written notes will be used. All interview data will be analysed using a framework approach [33, 34]. Data analysis will be supported by lay members of the research group, and output from the data analysis will be used to generate recommendations for the subsequent definitive trial.

### Trial oversight

Oversight is provided by the Trial Steering Committee (TSC) which comprises an independent chair and two independent members (including one patient representative). The TSC will also contribute to the final assessment for feasibility. Further details can be found in the acknowledgements. A separate data monitoring committee is not required due to the very low risk associated with the intervention; this function is be covered by the TSC.

### Assessment of feasibility

There are few examples of multi-centre RCTs in UK audiology clinics on which to inform the setting of pre-defined thresholds for specific feasibility outcomes. Upon the recommendation of the TSC, an adaptation of the feasibility assessment proposed by Thyer et al. [35] will be used to determine whether a fully powered randomised trial will be feasible. This will be assessed using both the quantitative and qualitative data gathered within the context of the present trial.

The feasibility study will be deemed successful if:

1. Eighty per cent of recruitment target is achieved.
2. Number of sites retained, number of site achieving recruitment target, study consent and retention rates and proposed sample sizes, indicate delivery of the full RCT is plausible within a 5-year study period.
3. Participants and clinicians report acceptability of the trial.

The Trial Management Group and TSC will consider the above criteria, resulting in one of the following outcomes:

1. Stop: definitive RCT not feasible;
2. Go: optimise design based on feasibility findings; apply for funding of a definitive RCT.

## Discussion

Without a standard care pathway across the UK for the management of tinnitus, we have chosen to undertake a feasibility trial to inform the design of a future definitive RCT to assess the effectiveness and cost-effectiveness of digital hearing aids in patients with tinnitus and hearing loss. We anticipate that this trial might present a number of methodological challenges as there have been relatively few previous large-scale RCTs engaging audiologists within UK audiology clinics; at least one large-scale RCT (of an investigational medicinal product) has reported the need to adjust procedures, recruitment and screening methods to meet targets [36]. Work still needs to be undertaken to help audiology clinics embed high-quality trials alongside their clinical practice. This present feasibility trial is a further step towards strengthening the culture of RCT research within this group of healthcare professionals.

The outcomes of this trial will provide the Trial Management Group and Trial Steering Committee with the data to determine if it is feasible to conduct a multicentre RCT comparing TAU and TAU plus digital hearing aids and to inform the best design of this future definitive RCT. This trial will also help to refine which outcomes would be most suitable for the definitive trial, and importantly to define which of the existing outcome instruments is most relevant to patients, and as such should be used as the primary outcome. This work is being carried out alongside work to define a core outcome set for early-phase clinical trials of sound-based interventions for chronic subjective tinnitus in adults [22, 23].

If the present trial shows that a definitive trial is feasible, the data gathered in the future trial can ultimately be used to facilitate evidence-based NHS commissioning and clinical practice in audiology and to ensure that provision of hearing aids for tinnitus will be informed by high-quality research evidence promoting equal access to management options and current technology for all tinnitus patients. We see the HUSH Feasibility Trial as an essential and valuable methodological step towards future research that will provide the needed evidence base for the clinical efficacy and cost-effectiveness of hearing aids as a tinnitus management strategy.

## Current trial status

Protocol version 1.2 4 September 2018. Recruitment opened on 15 October 2018 and will continue for a period of 12 months.

## Data Availability

Data sharing is not applicable to this article as no datasets were generated or analysed during the current study. The full trial protocol and all study documentation including information sheets, consent forms, questionnaires and other data collection forms will be made available with the publication of the final trial report. Final trial results will be written up and published by the trial team. Lay summaries will be sent to trial participants and relevant patient and public-facing groups. No professional writers will be used for any dissemination activities.

## Abbreviations

CRN: Clinical Research Network
EQ-5D-5L: Euroqol-5D-5L
GP: General practitioner
HRA: Health Research Authority
NCTU: Nottingham Clinical Trials Unit
NHS: National Health Service
NICE: National Institute for Health and Care Excellence
NIHR: National Institute for Health Research
NRES: National Research Ethics Service
QALY: Quality-adjusted life year
TSC: Trial Steering Committee
